# LncRNA XIST enhanced TGF-β2 expression by targeting miR-141-3p to promote pancreatic cancer cells invasion

**DOI:** 10.1042/BSR20190332

**Published:** 2019-07-02

**Authors:** Jianmin Sun, Yubao Zhang

**Affiliations:** Department of Hepatobiliary Pancreatic Surgery, Harbin Medical University Cancer Hospital, Harbin City 150040, Heilongjiang Province, P.R. China

**Keywords:** invasion, miR-141-3p, pancreatic cancer, TGF-β2, XIST

## Abstract

The level of expression of long non-coding RNA (LncRNA) X-inactive specific transcript (XIST) is up-regulated in pancreatic cancer (PC). However, the role of XIST in PC and the underlying mechanism are still unknown. The present study aimed to elucidate how XIST participates in PC and its potential target, miR-141-3p. We detected the XIST expression in PC tissues and cells by qRT-PCR. Cell proliferation was measured using a CCK8 kit, and the migration and invasion of cells was measured by Transwell assay. Silencing XIST and miR-141-3p was performed with transfection by Lipofectamine kit. Binding assay was conducted by luciferase reporter assay. Protein expression was examined by Western blot. These results indicate that (i) XIST is highly expressed in tumor tissues while miR-141-3p is down-regulated. (ii) Silencing XIST inhibits the pancreatic cell proliferation, migration and invasion. (iii) MiR-141-3p inhibitor alleviates the inhibitory effect by siXIST in PC cell lines. (iv) MiR-141-3p directly interacts with XIST and also negatively regulates transforming growth factor-β 2 (TGF-β2) expression. (v) Overexpression of XIST attenuates the inhibition of TGF-β2 expression by miR-141-3p. The conclusion, is that XIST could promote proliferation, migration and invasion of PC cells via miR-141-5p/TGF-β2 axis.

## Introduction

Pancreatic cancer (PC) is a highly malignant gastrointestinal tumor, which is still considered largely incurable. The PC progression is too rapid to be under effective control. In surprise, the 1-year relative survival rate of PC is approximately 20%, and more cruelly, the 5-year rate is 7% according to the American Cancer Society. Most patients survive less than 1 year after the first diagnosis [1,2]. In the past decade, even though the traditional management, including surgical resection, radiotherapy and chemotherapy, has continuously improved, there is still a rising trend in the incidence and mortality of PC. Now, PC is still a confusing issue to address it urgently. To explore the malignant progression of PC, especially the underlying mechanism, will provide a novel biomarker for the diagnosis and treatment.

Long non-coding RNAs (LncRNAs) are greater than 200 bp, without protein production, which play an important role to regulate gene expression through epigenetic and post-transcriptional levels. The X-inactive specific transcript (XIST) is an lncRNA encoded by the *XIST* gene, which mainly regulates X-chromosome inactivation of mammalian cells. It was reported that LncRNA XIST is highly expressed in several tumors, such as glioblastoma [3], breast and uterine cancer [4]. Knockdown XIST inhibits tumor growth by suppression of cell migration, invasion and as well as proliferation. *In vivo* studies also validated knocking out XIST gene suppressed tumor growth and improved survival in NICD mice [3]. As known, XIST is elevated in PC tissues, and its high expression promotes the progression of PC cells.

MicroRNAs are non-coding small RNAs of 18-25 nucleotides in length. They could knock down the corresponding mRNA expression by inducing the mRNA degradation or inhibiting mRNA translation by binding to the 3′-UTR. MiR-141-3p is abnormally expressed in a variety of tumors and is involved in a variety of cellular processes. MiR-141-3p exhibits both a pro- and tumor-suppressing effects in tumors. MiR-141-3p played as a cancer-promoting factor during the development of esophageal cancer [5]. It was reported that miR-141-3p could inhibit the expression of Kruppel-like factor 9 to promote prostate cancer cell progression [6]. However, in glioma cells and breast cancer, miR-141-3p is expressed as a tumor suppressor by regulating the activity of transcription factor 5 [7] and acting on p27/Kip1, CDK6, PR and Stat5a [8,9], respectively. In PC cells, miR-141 could directly match with 3′-UTR of MAPK4, thereby inhibiting cell proliferation [10]. Nevertheless, how miR-141-3p participates in PC cells and the underlying mechanism remains to be further investigated.

Transforming growth factor-β 2 (TGF-β2) is involved in the progression of pancreatic malignancies. Overexpression of TGF-β2 in PC is suggested to play a pivotal role in malignant progression by inducing angiogenesis, proliferation, immunosuppression and metastasis. Overexpression of TGF-β2 in the liver is associated with poor clinical prognosis of PC. In addition, TGF-β2, not TGF-β1, can also enhance Foxp3 expression in PC cells, resulting in immune escape [11]. However, the role of TGF-β2 in PC needs further investigation.

Our study aimed to examine how XIST and miR-141-3p participate in PC, and further to confirm whether miR-141-3p is the target of XIST. In addition, we explored the correlation and functions of XIST and TGF-β2 in PC invasion.

## Materials and methods

### Tumor tissue

Thirty pairs of tumor tissues and adjacent normal tissues from newly diagnosed PC patients were collected and the clinical information is shown in Table 1. Tissue samples were snap-frozen in liquid nitrogen and stored at −80°C. All cases were diagnosed by postoperative histopathological examiner. The patients with other major organic diseases were excluded. All the study was approved by the Ethics Committee of Harbin Medical University Cancer Hospital. All procedures performed in studies involving human participants were in accordance with the 1964 Helsinki Declaration and its later amendments or comparable ethical standards. The written informed consent was obtained from each individual patient.

**Table 1:** The information on 30 patients in the research

Group	Cases
**Age (years)**	
<45	4
≥45	26
**Sex**	
Female	13
Male	17
**Tumor size (cm)**	
<2	6
2–5	10
≥5	14
**Tumor stage**	
IA	3
IB	7
IIA	9
IIB	11
**Differentiation**	
Well differentiated	6
Moderately differentiated	8
Poorly differentiated	16
**Lymph node status**	
Negative	19
Positive	11

### Cell culture

PC cell line PANC-1 and human embryonic kidney cell HEK293T were purchased from the Cell Resource Center of Shanghai Institutes for Biological Sciences, Chinese Academy of Sciences. The cells were grown in Dulbecco’s Modified Eagle’s Medium (DMEM, Gibco, U.S.A.) supplemented with 10% fetal calf serum (Gibco, U.S.A.), 100 U/ml penicillin (HyClone), and 100 μg/ml streptomycin (HyClone) at 5% CO_2_, 37°C incubator with saturated humidity. All operations were performed under sterile conditions.

### Total RNA isolation

TRIzol assay (Invitrogen, Carlsbad, CA) was used to extract the total RNA according to the instructions. The quality and quantity of RNA were monitored by OD_260/280_. The RNA OD_260/280_ (1.8–2.0) was used for the experiments.

### qRT-PCR

RNA was reverse transcribed into cDNA according to the PrimeScript RT Reagent Kit (Takara, Japan). Quantitative PCR was performed using iQ™ SYBR Green Supermix (Bio-Rad, U.S.A.) in ABI Prism 7500 thermocycler. The qRT-PCR analysis of miR-141-3p was performed using the TaqMan MicroRNA Assay Kit (Applied Biosystems) kit, and chose U6 as an internal load. The primer sequences are as follows: XIST F: 5′- AGCTCCTCGGACAGCTGTAA-3′, R: 5′-CTCCAGATAGCTGGCAACC-3′. β-actin F: 5′-GTGGCCGAGGACTTTGATTG-3′, R: 5′-CCTGTAACAACGCATCTCATATT-3′. miR-141-3p F: 5′-TAACACTGTCTGGTAAAGATGG-3′. U6 F: 5′-CTCGCTTCGGCAGCACA-3′. The experiment was repeated three times. The relative expression of lncRNA XIST or miR-141-3p in each sample was calculated by ΔΔ*C*_t_ method.

### Cell proliferation

Cell proliferation was measured using a CCK8 kit (Nanjing Kaiji Biotechnology Development Co., Ltd. KGA317). Briefly, 1 × 10^4^ PANC-1 cells were seeded into 96-well plates at 100 μl/well. After incubating overnight, 10 μl of CCK solution was directly added into each well in a 96-well plate. After incubation for 2 h at 37°C, the absorbance at 450 nm of each well was monitored using VICTOR Multilabel Plate Reader (PerkinElmer, U.S.A.). Each sample was replicated for at least six times, the data were pooled and averaged.

### Cell migration and invasion

The cells were resuspended in serum-free medium and diluted to a concentration of 5 × 10^5^/ml. For cell migration assay, 200 μl of cell suspension was directly added to the upper chamber of the Transwell, and then 500 μl of medium containing 10% FBS was added to the lower chamber. For the cell invasion assay, 50 μl of Matrigel was added to the upper chamber of Transwell. One hundred microliters of cells in serum-free medium were placed on the upper chambers of a Transwell insert (8 μm pore size; BD Biosciences). Then 500 μl medium containing 10% FBS was placed into each lower chamber. The cells were finally fixed with 100% cold methanol, and stained with 0.05% Crystal Violet for 30 min. Five fields from each slides were randomly selected under inverted microscope. The images were captured, counted for analysis. The experiments were repeated individually for triplicates.

### Cell transfection

Cell transfection was performed with Lipofectamine 2000 kit (Invitrogen) according to the instructions. Briefly, PANC-1 cells were cultured in six-well plates at 1 × 10^6^/ml overnight, when the cells reached 70% confluence, the transfection of miR-141-3p mimic/inhibitor or miRNA-NC (negative control), siXIST or siRNA-NC were carried out, respectively. After 36-h culture, the cells were collected for experiment. The siRNA and the NC were synthesized by Shanghai Biotech with the following sequence: miR-141-3p mimic, 5′-UAACACU GUCUGGUAAAGAUGG-3′,miR-141-3p inhibitor, 5′-CCAUCUUUACCAGACAGUGUUA-3′, siXIST, 5′- GCUGACUACCUGAGAUUUATT-3′.

### Luciferase reporter assay

TargetScan software suggested that the 3′-UTR of lncRNA XIST and TGFb2 contains a putative binding site of miR-141-3p. Next, we synthesized the DNA fragment containing lncRNA XIS, its mutant fragment and TGFb2 fragment containing this site (Shanghai Shenggong), after ligation into the luciferase promoter vector psiCHECK2 dual luciferase vector (Promega, Madison, WI, U.S.A.). We co-transfected the plasmid and miRNA into HEK293T cells. After 48-h culture, luciferase activity was examined using a Dual-Luciferase® Reporter Assay System (Promega), the detection was monitored with GloMax® 20/20 Luminometer. The results were statistically analyzed.

### Western blot

The proteins were isolated with RIPA Lysis and Extraction Buffer (Thermo Fisher, U.S.A.) according to the manuals and protocols. The protein was subjected to SDS/PAGE gel for electrophoresis at 30 μg/well, and the protein was transferred to the PVDF membrane. The membrane was blocked with 5% skim milk/PBS for 2 h, and then incubated with the primary anti-TGF-β2 (Abcam) and internal control anti-GAPDH antibody (Abcam) at 4°C overnight. Then the membrane was incubated with the corresponding horseradish peroxidase-labeled secondary antibodies for 1 h at room temperature. Luminescence was developed using an ECL chemiluminescence detection kit (Kaki Bio). The intensities of the bands were quantitated using ImageJ software.

### Statistical analysis

The results represent the mean of three independent experiments, and the data are presented as the means ± standard deviation (SD) by SPSS 19.0. Comparisons between two groups were analyzed by Student’s *t* test, and multiple comparisons between groups were assessed by one-way ANOVA. A value of <0.05 was considered statistically significant.

## Results

### Expression of miR-141-3p and lncRNA XIST in tumor tissues

**Figure 1:**
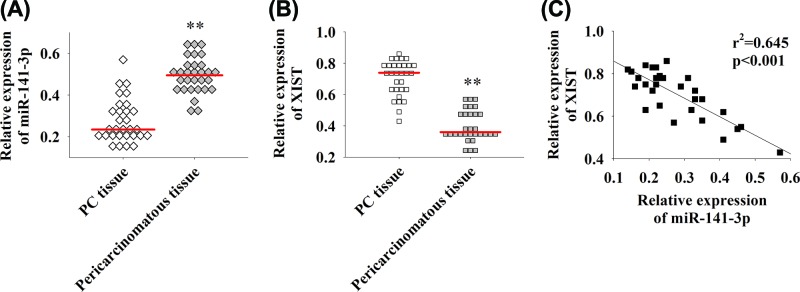
Expression of miR-141-3p and XIST in PC (**A**) qRT-PCR detection of miR-141-3p expression in tumor and the adjacent normal tissues. (**B**) qRT-PCR detection of lncRNA XIST expression levels in tumor and the adjacent normal tissues. (**C**) The correlation analysis between miR-141-3p and XIST expression. The red line represents the average, **P<0.01.

The expression of miR-141-3p and lncRNA XIST in PC and the adjacent normal tissues was detected by qRT-PCR. As shown in Figure 1A,B, it indicated that miR-141-3p is down-regulated in tumor tissues, while XIST is highly expressed in PC. The correlation analysis of miR-141-3p and lncRNA XIST showed they were negatively correlated (Figure 1C, r^2^ = 0.645, *P*<0.01). Therefore, the level expression of LncRNA XIST is up-regulated in PC and negatively correlated with miR-141-5p.

### Silencing of XIST inhibits proliferation, migration and invasion of PC cells

**Figure 2:**
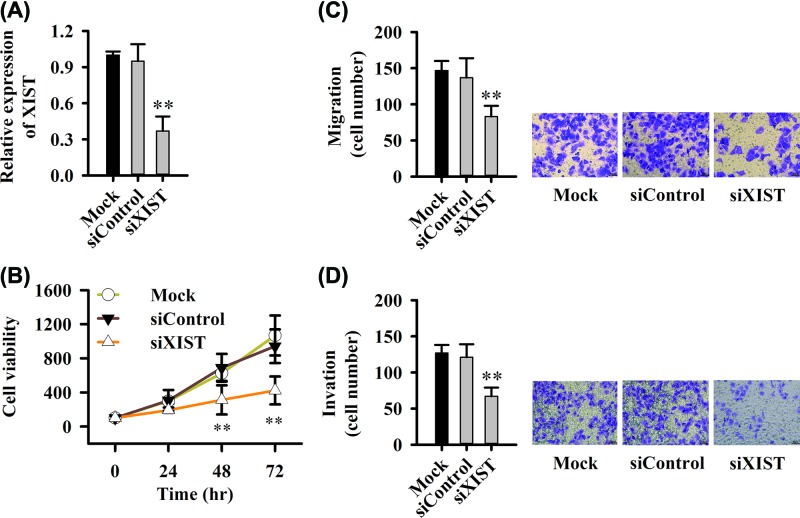
Silencing XIST inhibits proliferation, migration and invasion of PC cells (**A**) siRNA silences the expression of lncRNA XIST. (**B**) siXIST inhibited the cell proliferation. (**C**) Transwell assay detects the inhibition of cell migration by siXIST. (**D**) Transwell detects the inhibition of cell invasion by siXIST. Six fields of view in migration and invasion assay were randomly counted, **P<0.01.

To excavate the function of XIST in PC cell lines, we transfected cells with siXIST, siControl or Mock. We found siXIST could significantly decrease the expression of XIST (Figure 2A), and siXIST inhibited cell proliferation (Figure 2B). The migration and invasion was significantly inhibited after siXIST transfection (Figure 2C,D). Therefore, XIST is also a pro-cancerous factor in PC cells, and silencing its expression can inhibit tumor cellular processes, such as the activities of proliferation and invasion.

### MiR-141-3p inhibitor reverses the inhibitory effect by siXIST on proliferation, migration and invasion of PC cell lines

**Figure 3:**
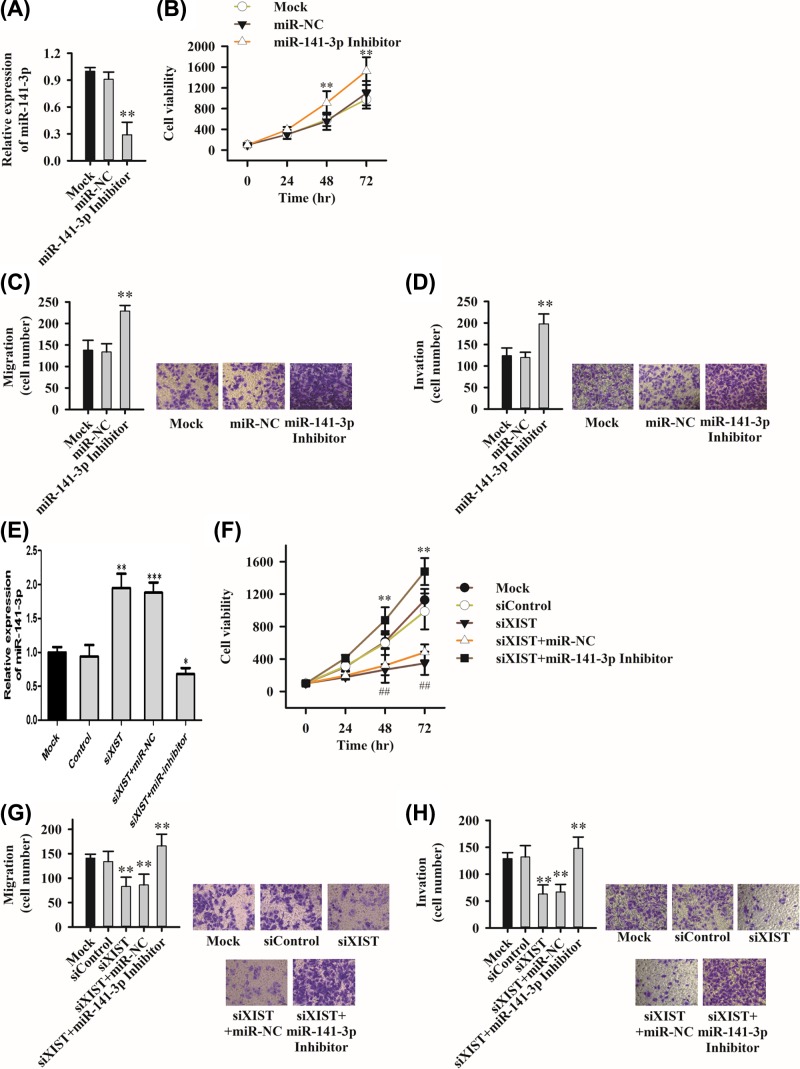
MiR-141-3p inhibitor reverses the inhibitory effect of siXIST on proliferation, migration and invasion of PC cell lines (**A**–**D**) Knockdown of miR-141-3p expression affects cell proliferation (B), migration (C) and invasion (D). The effect of (**E**–**H**), miR-141-3p inhibitor and siXIST co-transfection on cell proliferation (F), migration (G) and invasion (H). Six fields of view in migration and invasion assay were randomly counted, *P＜0.05,**P＜0.01, ***P＜0.001.

To investigate the function of miR-141-3p in PC cell lines, PC cells transfected with miR-141-3p inhibitor and its corresponding controls miR-NC and Mock, respectively. The level expression of miR-141-3p was indeed down-regulated after transfection of miR-141-3p inhibitor (Figure 3A). Meanwhile the cell proliferation (Figure 3B), migration (Figure 3C) and invasion (Figure 3D) were promoted. To study the interaction between miR-141-3p and XIST, PC cells were co-transfected with siXIST and miR-141-3p inhibitor. The results showed siXIST increased the miR-141-3p expression (Figure 3E). Notably, there was a reversed effect among the siXIST cell on proliferation (Figure 3F), migration (Figure 3G) and invasion (Figure 3H) when miR-141-3p inhibitor was added, suggesting that miR-141-3p and XIST interacted and exhibited functional negatively.

### MiR-141-3p directly interacts with XIST

**Figure 4:**
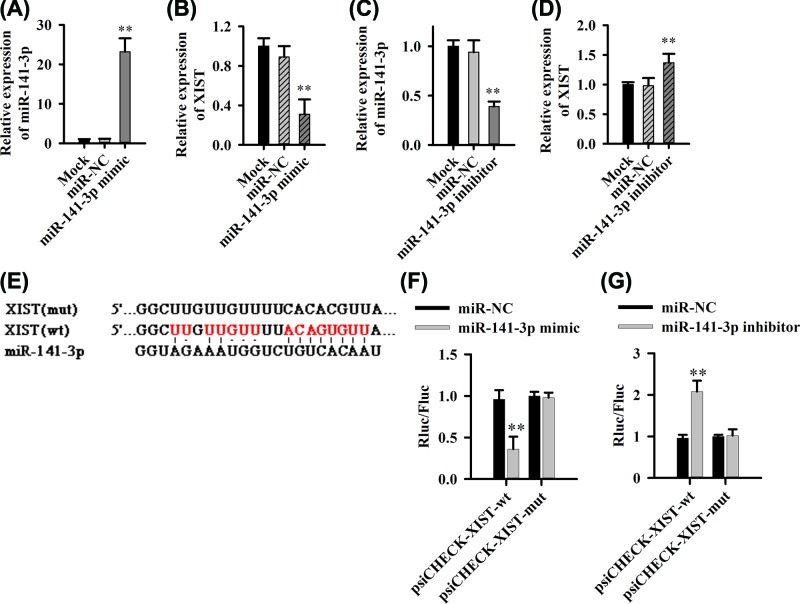
MiR-141-3p can directly bind with XIST (**A,B**) The effect of up-regulation of miR-141-3p on XIST expression. (**C,D**) The down-regulation of miR-141-3p expression on XIST expression. (**E**) miR-141-3p and XIST binding site prediction. (**F,G**) Luciferase dual reporter system detects the direct effect of miR-141-3p and XIST, **P<0.01.

The aforementioned studies suggested that there might be interactions between miR-141-3p and XIST. Therefore, we further excavate the influence of up-regulated miR-141-3p expression on high-level expression of XIST. The intracellular miR-141-3p levels were increased when cells transfected with miR-141-3p mimics (Figure 4A) while XIST expression was down-regulated (Figure 4B). Meanwhile, when cells transfected with miR-141-3p inhibitor, intracellular miR-141-3p levels were reduced (Figure 4C) and XIST expression was up-regulated (Figure 4D), these data further validated the negative interaction of miR-141-3p and XIST.

TargetScan.org predicts that miR-141-3p and XIST may directly interact (Figure 4E). To prove whether there is a direct interaction between these two factors, we used a dual fluorescence reporter system. We found that miR-141-3p mimic acts directly on the wild-type XIST target sequence, causing a decrease in fluorescence (Figure 4F). However, the fluorescence of the mutant XIST sequence was not affected (Figure 4F). Similarly, the miR-141-3p inhibitor could increase the fluorescence of the wild-type XIST sequence (Figure 4G), whereas there was no influence on mutant XIST sequence (Figure 4G).

### MiR-141-3p negatively regulates TGF-β2 expression

**Figure 5:**
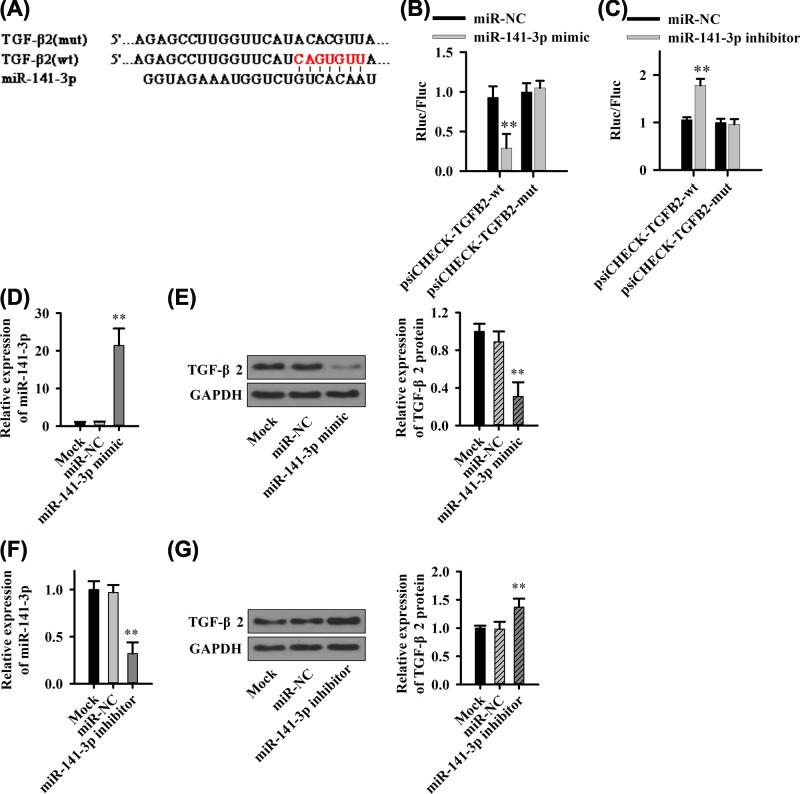
MiR-141-3p regulates TGF-β2 expression (**A**) miR-141-3p and TGF-β2 binding site prediction. (**B,C**) Luciferase dual reporter system to detect the direct effect of miR-141-3p and TGF-β2. (**D**) Up-regulation of miR-141-3p expression (**E**) Effect of TGF-β2 expression. (**F**) miR-141-3p expression down-regulates TGF-β2 expression (**G**). **P<0.01.

The previous studies have shown that XIST might accelerate the process of TGF-β-induced EMT by regulating the miR-367/141-ZEB2 axis in NSCLC. However, whether XIST has a regulatory effect on the function of TGF-β in PC remains unknown. To address this issue, we used software to predict that miR-141-3p and TGF-β2 might interact (Figure 5A). The dual fluorescence reporter system was used to validate the interaction between miR-141-3p and TGF-β2. The results indicate miR-141-3p mimic could cause the fluorescence decrease by matching directly with sequence of wild-type TGF-β2 (Figure 5B), but not affect the mutant one (Figure 5B). Similarly, the miR-141-3p inhibitor enhanced the fluorescence of the wild-type TGF-β2 (Figure 5C) whereas it did not exert an influence on the mutant one (Figure 5C).

We further examined the effect of miR-141-2p on TGF-β2 protein levels. After transfection of miR-141-3p mimic or inhibitor, miR-141-3p expression was up-regulated (Figure 5D) or down-regulated (Figure 5F) and TGF-β2 protein expression was down-regulated (Figure 5E) or up-regulated (Figure 5G), respectively.

### Overexpression of XIST attenuates the inhibition of TGF-β2 expression by miR-141-3p

**Figure 6:**
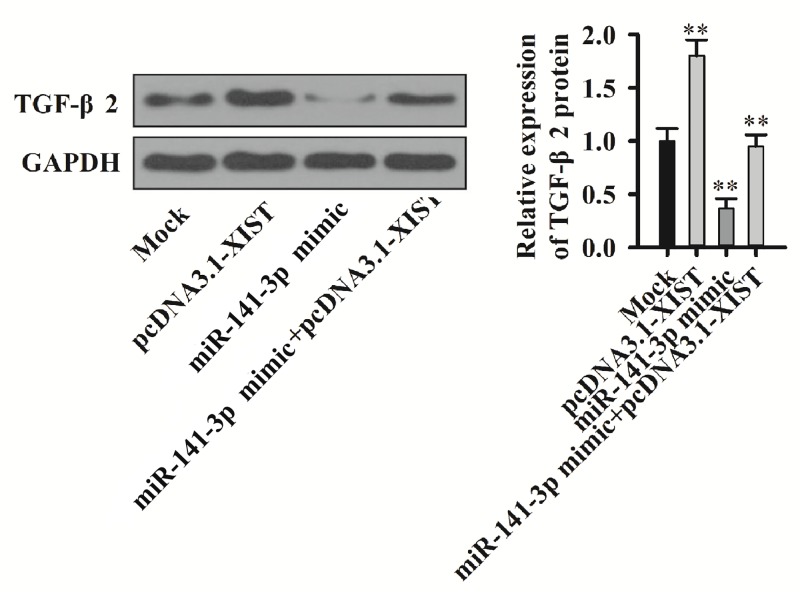
XIST binds miR-141-3p to regulate TGF-β2 expression TGF-β2 protein expression detection by Western blot and quantitation in miR-141-3p mimic and pcDNA3.1-XIST co-transfection cells.

After co-transfection of cells with miR-141-3p mimic and pcDNA3.1-XIST, we found that the TGF-β2 protein expression was inhibited by miR-141-3p, while overexpression of XIST reversed down-regulation on TGF- β2 by miR-141-3p (Figure 6). These results indicate XIST and miR-141-3p interactions affect the TGF-β2 expression in PC cell lines.

## Discussion

In this research, the primary objective was to investigate the relationship between lncRNA XIST and miR-141-3p in human PC and to explore the underlying mechanisms that influence the cellular process in PC cells. We found that miR-141-3p has a lower level expression in tumor tissues while XIST is highly expressed in tumor tissues, XIST is negatively correlated with miR-141-5p. Knockdown of XIST inhibits proliferation, migration and invasion of PC cells while miR-141-3p inhibitor reverses the suppression by siXIST on these effects. Further, we confirmed that miR-141-3p directly interacts with XIST; miR-141-3p negatively regulates TGF-β2 expression. Overexpression of XIST attenuates the inhibition of TGF-β2 expression by miR-141-3p.

LncRNA XIST is an LncRNA, participating in X-chromosome inactivation process. There is increasing evidence that XIST is closely associated with the development of tumors. In PC patients, high XIST expression always reflects worse clinical stage. miR-141 regulated MAPK4 in PC cells, inhibiting its expression and inhibiting cell proliferation [10]. Our study demonstrated there was a negative correlation between the expression of XIST and miR-141-3p in PC tissues. Silencing XIST inhibits PC progression, which is consistent with other reports that XIST appears to be a carcinogenic factor in PC.

TGF-β signaling pathway is involved in the progression of PC critically [12]. There are three major subtypes of TGF-β: TGF-β1, TGF-β2 and TGF-β3, which overlap in functions and all three subtypes are expressed in PC [13]. Currently, the research on TGF-β mainly only focuses on TGF-β1, which exhibits distinguished roles in different stages of tumor development. In the early stage of normal epithelial cells and tumor development, TGF-β1 suppresses the tumor growth. While, in the later stage, TGF-β1 mediates tumor cell proliferation, invasion and metastasis [14–16]. Although TGF-β1 and TGF-β2 exist in highly homologous sequence, *in vivo* experiments demonstrated that they play huge different functions [17,18]. TGF-β2 was first thought to be a cytokine secreted by tumor cells, which can inhibit human immune cells [19,20] to help tumor cells escape the surveillance of the immune system and promote cell migration, infiltration and metastasis [21]. Studies have shown that TGF-β2 expression is in tumor [13] or plasma [22], indicative of important roles of TGF-β2 in malignant cancers. In our present study, through the dual fluorescent reporter system, the results verified that miR-141-3p binds directly to XIST and inhibits each other. TGF-β2 is another target mRNA of miR-141-3p negatively regulating TGF-β2 in PNCA-1 cells. Overexpression of XIST attenuated the inhibition of TGF-β2 expression by miR-141-3p due to mutual feedback among the three factors. The results at the cellular level showed that XIST promotes PC cell progression via miR-141-5p/TGF-β2 axis. These results may provide the therapeutic target, through manipulating the small molecular of miR-141-3p, to govern the PC cell behavior, especially of cell invasion in the clinic in future.

Therefore, overexpression of XIST promotes proliferation, migration and invasion of PC cells via miR-141-5p/TGF-β2 axis.

## Abbreviations

CCK8: Cell Counting kit-8
CDK6: Cyclin-dependent kinase-6
EMT: Epithelial-mesenchymal transition
Foxp3: Forkhead box P3
LncRNA: long non-coding RNA
MAPK4: Mitogen-activated protein kinase-4
NC: negative control
NSCLC: non-small cell lung cancer
OD: optical density
PC: pancreatic cancer
PR: progesterone receptors
RIPA: Radio immunoprecipitation assay
TGF-β: transforming growth factor-β
XIST: X-inactive specific transcript

## References

[1] Jemal A., Siegel R., Ward E., Hao Y., Xu J., Murray T. (2008) Cancer statistics, 2008. CA Cancer J. Clin. 58, 71–96 10.3322/CA.2007.0010 18287387

[2] Cardenes H.R., Chiorean E.G., Dewitt J., Schmidt M. and Loehrer P. (2006) Locally advanced pancreatic cancer: current therapeutic approach. Oncologist 11, 612–623 10.1634/theoncologist.11-6-612 16794240

[3] Yao Y., Ma J., Xue Y., Wang P., Li Z., Liu J. (2015) Knockdown of long non-coding RNA XIST exerts tumor-suppressive functions in human glioblastoma stem cells by up-regulating miR-152. Cancer Lett. 359, 75–86 10.1016/j.canlet.2014.12.051 25578780

[4] Ren C., Li X., Wang T., Wang G., Zhao C., Liang T. (2015) Functions and mechanisms of long noncoding RNAs in ovarian cancer. Int. J. Gynecol. Cancer 25, 566–569 10.1097/IGC.0000000000000413 25756403

[5] Jin Y.Y., Chen Q.J., Xu K., Ren H.T., Bao X., Ma Y.N. (2016) Involvement of microRNA-141-3p in 5-fluorouracil and oxaliplatin chemo-resistance in esophageal cancer cells via regulation of PTEN. Mol. Cell. Biochem. 422, 161–170 10.1007/s11010-016-2816-9 27644195

[6] Li J.Z., Li J., Wang H.Q., Li X., Wen B. and Wang Y.J. (2017) MiR-141-3p promotes prostate cancer cell proliferation through inhibiting Kruppel-like factor-9 expression. Biochem. Biophys. Res. Commun. 482, 1381–1386 10.1016/j.bbrc.2016.12.045 27956179

[7] Wang M., Hu M., Li Z., Qian D., Wang B. and Liu D.X. (2017) miR-141-3p functions as a tumor suppressor modulating activating transcription factor 5 in glioma. Biochem. Biophys. Res. Commun. 490, 1260–1267 10.1016/j.bbrc.2017.05.179 28595907 PMC5759330

[8] Uhlmann S., Zhang J.D., Schwager A., Mannsperger H., Riazalhosseini Y., Burmester S. (2010) miR-200bc/429 cluster targets PLCgamma1 and differentially regulates proliferation and EGF-driven invasion than miR-200a/141 in breast cancer. Oncogene 29, 4297–4306 10.1038/onc.2010.201 20514023

[9] Finlay-Schultz J., Cittelly D.M., Hendricks P., Patel P., Kabos P., Jacobsen B.M. (2015) Progesterone downregulation of miR-141 contributes to expansion of stem-like breast cancer cells through maintenance of progesterone receptor and Stat5a. Oncogene 34, 3676–3687 10.1038/onc.2014.298 25241899 PMC4369481

[10] Maruyama R., Suzuki H., Yamamoto E., Imai K. and Shinomura Y. (2012) Emerging links between epigenetic alterations and dysregulation of noncoding RNAs in cancer. Tumour Biol. 33, 277–285 10.1007/s13277-011-0308-9 22219034

[11] Hinz S., Pagerols-Raluy L., Oberg H.H., Ammerpohl O., Grussel S., Sipos B. (2007) Foxp3 expression in pancreatic carcinoma cells as a novel mechanism of immune evasion in cancer. Cancer Res. 67, 8344–8350 10.1158/0008-5472.CAN-06-3304 17804750

[12] Truty M.J. and Urrutia R. (2007) Basics of TGF-beta and pancreatic cancer. Pancreatology 7, 423–435 10.1159/000108959 17898532

[13] Friess H., Yamanaka Y., Buchler M., Ebert M., Beger H.G., Gold L.I. (1993) Enhanced expression of transforming growth factor beta isoforms in pancreatic cancer correlates with decreased survival. Gastroenterology 105, 1846–1856 10.1016/0016-5085(93)91084-U 8253361

[14] Bierie B. and Moses H.L. (2006) Tumour microenvironment: TGFbeta: the molecular Jekyll and Hyde of cancer. Nat. Rev. Cancer 6, 506–520 10.1038/nrc1926 16794634

[15] Derynck R. and Akhurst R.J. (2007) Differentiation plasticity regulated by TGF-beta family proteins in development and disease. Nat. Cell Biol. 9, 1000–1004 10.1038/ncb434 17762890

[16] Hata A., Shi Y. and Massague J. (1998) TGF-beta signaling and cancer: structural and functional consequences of mutations in Smads. Mol. Med. Today 4, 257–262 10.1016/S1357-4310(98)01247-7 9679244

[17] Shull M.M., Ormsby I., Kier A.B., Pawlowski S., Diebold R.J., Yin M. (1992) Targeted disruption of the mouse transforming growth factor-beta 1 gene results in multifocal inflammatory disease. Nature 359, 693–699 10.1038/359693a0 1436033 PMC3889166

[18] Sanford L.P., Ormsby I., Gittenberger-de Groot A.C., Sariola H., Friedman R., Boivin G.P. (1997) TGFbeta2 knockout mice have multiple developmental defects that are non-overlapping with other TGFbeta knockout phenotypes. Development 124, 2659–2670 9217007 10.1242/dev.124.13.2659 PMC3850286

[19] Fontana A., Hengartner H., de Tribolet N. and Weber E. (1984) Glioblastoma cells release interleukin 1 and factors inhibiting interleukin 2-mediated effects. J. Immunol. 132, 1837–1844 6607949

[20] Kuppner M.C., Hamou M.F., Sawamura Y., Bodmer S. and de Tribolet N. (1989) Inhibition of lymphocyte function by glioblastoma-derived transforming growth factor beta 2. J. Neurosurg. 71, 211–217 10.3171/jns.1989.71.2.0211 2545842

[21] Massague J. (2008) TGFbeta in cancer. Cell 134, 215–230 10.1016/j.cell.2008.07.001 18662538 PMC3512574

[22] Bellone G., Smirne C., Mauri F.A., Tonel E., Carbone A., Buffolino A. (2006) Cytokine expression profile in human pancreatic carcinoma cells and in surgical specimens: implications for survival. Cancer Immunol. Immunother. 55, 684–698 10.1007/s00262-005-0047-0 16094523 PMC11031060

